# Systematic identification of phenotypically enriched loci using a patient network of genomic disorders

**DOI:** 10.1186/s12864-016-2569-6

**Published:** 2016-03-15

**Authors:** Armando Reyes-Palomares, Aníbal Bueno, Rocío Rodríguez-López, Miguel Ángel Medina, Francisca Sánchez-Jiménez, Manuel Corpas, Juan A. G. Ranea

**Affiliations:** Universidad de Málaga, Andalucía Tech, Departamento de Biología Molecular y Bioquímica, Facultad de Ciencias, and IBIMA (Biomedical Research Institute of Málaga), E-29071 Málaga, Spain; CIBER de Enfermedades Raras (CIBERER), E-29071 Málaga, Spain; The Genome Analysis Centre, Norwich Research Park, Norwich, NR4 7UH UK; Present address: The European Molecular Biology Laboratory Heidelberg, 69117 Heidelberg, Germany

## Abstract

**Background:**

Network medicine is a promising new discipline that combines systems biology approaches and network science to understand the complexity of pathological phenotypes. Given the growing availability of personalized genomic and phenotypic profiles, network models offer a robust integrative framework for the analysis of "omics" data, allowing the characterization of the molecular aetiology of pathological processes underpinning genetic diseases.

**Methods:**

Here we make use of patient genomic data to exploit different network-based analyses to study genetic and phenotypic relationships between individuals. For this method, we analyzed a dataset of structural variants and phenotypes for 6,564 patients from the DECIPHER database, which encompasses one of the most comprehensive collections of pathogenic Copy Number Variations (CNVs) and their associated ontology-controlled phenotypes. We developed a computational strategy that identifies clusters of patients in a synthetic patient network according to their genetic overlap and phenotype enrichments.

**Results:**

Many of these clusters of patients represent new genotype-phenotype associations, suggesting the identification of newly discovered phenotypically enriched *loci* (indicative of potential novel syndromes) that are currently absent from reference genomic disorder databases such as ClinVar, OMIM or DECIPHER itself.

**Conclusions:**

We provide a high-resolution map of pathogenic phenotypes associated with their respective significant genomic regions and a new powerful tool for diagnosis of currently uncharacterized mutations leading to deleterious phenotypes and syndromes.

**Electronic supplementary material:**

The online version of this article (doi:10.1186/s12864-016-2569-6) contains supplementary material, which is available to authorized users.

## Background

Genomic Structural Variations are one of the main sources of human genome variation. Copy Number Variations (CNVs) naturally occur in the genome of healthy individuals [1, 2], some of them leading to disease [3]. CNVs consist of thousands to millions of bp deletions, duplications, insertions or inversions, recurrent in the population either by inheritance or spontaneous occurrence (*de novo*) [4]. Although the discovery of CNVs was relatively recent, a plethora of genetic association studies have been carried out to understand their evolutionary [5], functional [6] and phenotypic effects [4]. It has been estimated that two genomes can differ approximately about 0.4 % due to CNVs [7] and that these variations have a considerable impact on human health. Several known chromosome imbalances causing complex genomic disorders have been characterized by different medical conditions such as developmental [8, 9], neuropsychiatric [10–12], cancer [13], autoimmune diseases [14] and idiopathic learning disability [15]. However, recent genome wide association studies suggest that the lack of data for individual’s medical records is an important limitation to fully understand the genetic basis for many genomic disorders [16, 17]. Initiatives such as the Personal Genomes Project (PGP) [18], Genomics England (http://www.genomicsengland.co.uk/) and the Precision Medicine program [19] aim to provide descriptive records and associated genomic data accessible for research. These datasets, however, are still unavailable or pose different challenges when looking into genetic association studies: e.g., lack of sizable data (e.g., PGP) or too restrictive access (e.g., Genomics England). These shortcomings may encourage genetic association studies to oversimplify complex phenotypic profiles of individuals, focusing on the most representative clinical features [20]. This makes it more difficult to characterize pathophysiological associations between clinical features observed in studied individuals [20]. New systematic and standardized methods are thus required that make use of limited accessible clinical genotype and phenotype profiling datasets to enhance our understanding of the genetic impact of CNVs on human health [21]. The present work uses individual clinical and genetic information stored in the DECIPHER Database [21], a database of sub-microscopic chromosome abnormalities (deletions and duplications) observed in clinic with a potential pathogenic association. Data currently stored by DECIPHER add up to more than 45,000 patients (march 2015), of which more than 10,000 have given consent to share their medical data [22] under an ethically regulated data access protocol. We focus our study on a subset of these data of 9,186 unbalanced CNVs from 6,564 patients that included a heterogeneous set of pathophenotypes, including developmental delay, intellectual disability and congenital malformations. Network analyses has been used in previous studies to characterize affected pathways by CVNs in cancer [23]. Here we applied network medicine approaches, phenotypic enrichment analyses and genetic association studies to build patient networks to explore the similarities between reported genetic microvariations (CNVs) and pathological phenotypes. We represented patients as nodes connected with edges to other patients whose CNVs overlap. Our resulting networks allowed the systematic identification of genetically related clusters of patients by finding cliques [24, 25]. A phenotypic enrichment analysis of patient clusters was performed to identify overrepresented phenotypes for each cluster. We named *Phenotypically Enriched Locus* (PEL) an affected genomic location showing significant associations with phenotypes. Significant genotype-phenotype associations were retrieved through the comparison of patients (cases) and healthy (controls) datasets, using a case–control association analysis. The combined use of these methods allowed us to build a high-resolution genotype-phenotype map that identifies a) already known, b) potentially novel genomic disorders and c) the additive phenotypic effects found in some proximal structural variations.

## Methods

### Case and control datasets

#### Cases

Rare CNVs (frequency of <1 %) from patients with low prevalent genomic disorders were downloaded from DECIPHER database (08/05/2014; http://decipher.sanger.ac.uk/) through its Data Access Agreement. This dataset contains genotype-phenotype annotations of consented DECIPHER patients, including chromosome locations, type of structural variant (gain or loss), mode of inheritance (de novo, inherited from unaffected parent, inherited from affected parent and unknown) and clinical phenotypes observed by expert physicians. When available, patients in DECIPHER are assigned phenotypes from the Human Phenotype Ontology (HPO), a standard controlled vocabulary of pathological terms [26]. Patients not annotated with HPO phenotypes were removed from our study. To reduce heterogeneity among collected patient data from DECIPHER, we only selected CNVs originated from array CGH technology, which corresponds to the majority of the database’s genotypic data. A final dataset of 6,564 patients with 9,186 CNVs presenting 1,860 non-redundant HPO terms was chosen for this study (Additional file 1: Table S1). Access to DECIPHER genomic coordinates of chromosomal microdeletions, microduplications and associated phenotypes were obtained through a Data Access Agreement. All data shared by the DECIPHER database have signed a consent form obtained by the submitting clinician. Those who carried out the original analysis and collection of the data bear no responsibility for the further analysis or interpretation of it by the Recipient or its Registered Users.

#### Controls

CNVs from healthy individuals were retrieved from the Database of Genomic Variants (DGV, http://dgv.tcag.ca/) [27], which provides a curated collection of human structural variations in control data from multiples studies. DGV offers information about CNVs of individual samples such as chromosome locations, type of structural variation (gain or loss) and reference (PubMed ID) of the study and the platform used in the analysis. The control structural variants dataset ("*GRCh37_hg19_variants_2014-10-16.txt*") was downloaded from DGV. This dataset combines CNV data from diverse studies. Using DGV as the control dataset has the caveat that it does not distinguish unrelated from related samples (i.e., the same patient CNV retrieved from different studies). Although in practice this overrepresentation of the same patient may seldom happen, it may still overestimate the number of so-called independent CNVs, affecting our final results. This overestimation of the frequency of CNVs in controls drove us to make a stricter assessment of the statistical significance of our predicted pathogenic CNVs. The types of effects this inflation of non-pathogenic CNVs may cause include an increase of the number of false negatives (i.e. true pathogenic CNVs that overlap with an over-estimated number of control CNVs) and a reduction of the number of false positives (i.e. false pathogenic CNVs overlapping with an over-estimated number of control CNVs). Therefore, we have considered CNVs from DGV only as a quantitative control for preventing misclassifications of CNVs as pathogenic.

### Building the genotype-based patient network

**Fig. 1:**
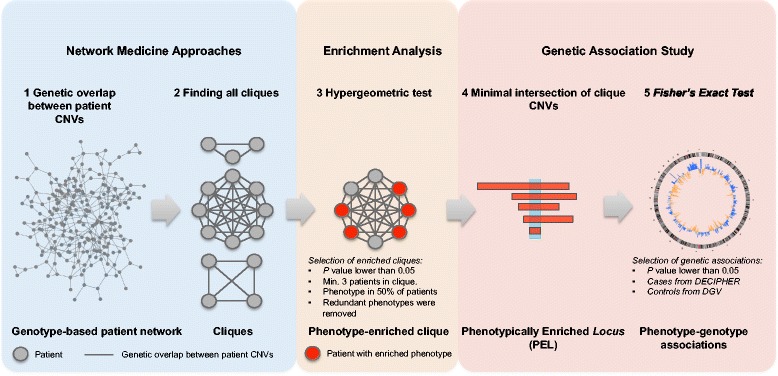
Workflow used to carry out the present study. More details can be found out in Methods

We designed a workflow to systematically identify all the existing genotype-phenotype associations in the case dataset (Fig. 1). First, the overlap between patient CNVs belonging to the same class (either gains or loses) was computed using the GRCh37/hg19 reference genome. For the purposes of this study, we assumed that two patient CNVs overlap if at least they share one common base pair. The resulting genetic relationships were used to build the network, where nodes are patients and edges represent the overlap between patient CNVs (Fig. 1).

### Clustering of patients using cliques

Finding all the k-cliques associated with each patient provides all complete graphs from the resulting genotype-based patient network. These cliques correspond to sets of variable numbers of nodes where all are connected to all by edges [24]. To find all the cliques associated with each node from the patient network, we used the algorithm of the function “cliques_containing_node” available in the Python package named NetworkX. The minimum size of cliques was limited to three patients (*k* nodes ≥ 3) but no limitation was applied to maximum size clique detection. We then merged into one clique all those containing identical sets of patients with the aim of getting a unique list of cliques resulting from the patient network. This list of unique cliques is of high interest for our approach because it allows the systematic identification of the whole set of patients sharing similar genotypes by mining directly the clusters of the network. Taking into account that CNV lengths can be very variable across the case population, a large patient CNV can overlap with other patient CNVs at different genomic regions. These complex interactions in the patient network imply that some cliques might not necessarily represent a cluster of patients where all their CNV overlap. Thus, we selected only those cliques that were fully represented by patients with mutations on the same genomic region. The resulting cliques were used as the list of clusters of patients to be used for downstream analyses, i.e., phenotype enrichment analysis.

### Phenotype enrichment analysis

The Human Phenotype Ontology (HPO) was used as a relational graph to identify common phenotypes among all the clique patients. The hierarchical organization of HPO terms (phenotypes) by parent–child relationships allows the detection of phenotype enrichments when their annotations co-occur at the same ontological level. We used this relational graph to detect the common phenotypes in a given cluster –or clique– of patients. To systematically assess the phenotype significance in each clique, we used a hypergeometric test and adjusted the *P-values* using Bonferroni. This test compares the frequency of every HPO term in each clique (number of observed cases in the sample) against their frequency in the whole dataset of annotated patients (observed cases in the population). To carry out this test, we used the number of individuals per clique as the sample size, the number of patients in the samples presenting a phenotype as the observed cases, and the total number of patients in DECIPHER database presenting the phenotype as the population size. We selected clique-phenotype enrichment associations by applying three different thresholds: 1) *P* < 0.05 from hypergeometric test, 2) counting at least three patients annotated with the enriched phenotype, and 3) if at least 50 % of the patients in the clique are annotated with the enriched phenotype. Once this selection process ended, we found that many of these cliques were enriched with HPO terms that are closely related in the ontology (i.e. parent–child relationship), producing some redundancy that does not add information. In those cases, redundancies were removed by selecting the most significant (lowest *P-values*) HPO terms as the representative ones.

### Characterizing phenotypically enriched *loci* (PELs)

We defined a phenotypically Enriched Locus (PEL) as the minimal common intersection among all the CNVs of patients in every clique that is significantly enriched with phenotypes (Fig. 1). We studied PELs’ incidence in patients (cases) by comparing them to a healthy population (control). Their statistical significance was assessed using a Fisher's exact test from a contingency table. This table consisted of a) the number of patients in a PEL associated with an enriched phenotype versus the total number of observed cases with that particular phenotype, and b) the number of healthy individuals –or samples from DGV dataset– with structural variants overlapping to this PEL versus the rest of observed controls (i.e., healthy population). We checked overlaps between PELs and individual control CNVs that overlapped at least 1 bp. After applying the Fisher's exact test, the *P-values* were adjusted using Benjamini & Hochberg and only those PEL sites with *P* < 0.05 were considered. This procedure allowed us to calculate the statistical significance of associations between enriched phenotypes (HPO-term) and a PEL compared to frequency of CNVs from the healthy population on the same locus. Finally, the penetrance of enriched phenotypes for each *locus* was calculated as the proportion of individuals showing the enriched phenotype –cases- over the healthy population –control-, by using a similar approach to the one recently published by Cooper et al. [8, 28].

### Randomization analysis on case and control datasets

Five randomization analyses were designed to test different null hypotheses: (i) Arbitrarily selected CNVs from the control dataset without replacement and it was used to test if the frequency of detected PELs is lower than from a case population (DECIPHER) when using CNVs from a healthy population (DGV). This randomization analysis was named “random patient CNVs from DGV”. (ii) The second type of randomized case dataset was generated from arbitrary genomic regions while keeping the CNV length distribution and chromosome frequencies from the case dataset and it was named “random patient CNV location”. This randomized dataset was used to test if the frequency of detected PELs is lower when individual case CNVs are randomly distributed across the genome compared to real patient CNVs from DECIPHER. (iii) A similar approach as mentioned above was used to generate the third type of randomized dataset but using the control dataset (DGV) instead of the case dataset. This randomization analysis, named “random control CNV location”, was used to test if the frequency of PELs is lower when individual control CNVs are randomly distributed across the genome compared to real CNVs from DGV. (iv) The fourth type of randomization analysis was carried out by randomly shuffling the patient-CNVs relations (named as “rewiring patient-CNV”) to test if the frequency of PELs is lower when using arbitrary phenotype-genotype relationships. (v) Finally, randomized case datasets were built using arbitrary phenotype descriptions of patients while keeping the phenotype frequency, to ensure that the representativeness of phenotypes from the real data is preserved. This randomization analysis was used to test that the frequency of detected PELs is lower using arbitrary phenotype descriptions for patients. We carried out one thousand randomization experiments for each randomized dataset and counted the number of PELs as well as the significances derived from the phenotypic enrichment analysis (*P-values* < 0.05, hypergeometric test) and genetic association study (*P-values* < 0.05, Fisher’s exact test).

## Results and discussion

### Phenotypic and genotypic features of patient population

**Fig. 2:**
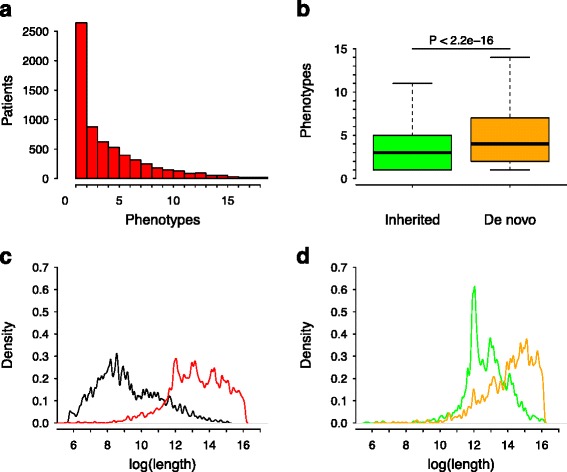
CNV length vs. phenotype relationships. **a** Histogram for the number of phenotypes observed in DECIPHER patients. **b** Boxplots of the number of phenotypes observed in patients showing inherited or *de novo* CNVs (because this CNV was absent in parents). For this plot, we only took into account those patients for whom only one CNV was detected. **c** Length CNV distributions for control (black line) and case (red line) populations. **d** Length CNV distributions in cases for *de novo* CNVs (orange line) and inherited CNVs by parents that do not manifest any pathogenic phenotype (green line)

The subset of 6,564 patients from the DECIPHER database used in this study includes the CNVs and clinical features (i.e., HPO phenotypic terms) observed by expert physicians in these patients. Table 1 summarizes the data analyzed for case (patients) and control (healthy population) datasets. The distribution of different phenotypes (HPO terms) associated with patients (Fig. 2a) showed that almost half of patients were annotated with just one HPO term, while the remaining cases showed more complex phenotypic profiles with two or more associated terms. The distributions of *de novo* and inherited patients were explored based on the complexity of their phenotypic profiles (Fig. 2b). It is observed that the *de novo* CNVs show a significant (*P* < 2.2E-16, Mann–Whitney *U* test) bias toward more complex –or diverse– phenotype profiles than the inherited group (Fig. 2b). The distribution of CNV lengths in patients is biased toward higher lengths as compared with those of control CNVs, something that should be expected if clinicians remove the non-pathological CNVs (Fig. 2c). Within the observed patient dataset, those including *de novo* CNVs showed the highest average length compared to the inherited set (Fig. 2d). These results suggest a positive relationship between CNV length and the complexity of annotated patient phenotypes. This is not a surprising observation, since larger CNVs affect more genes in the genome, producing an additive effect to observed clinical features.

**Table 1:** Population dataset descriptions

	All patients	Cases	Control
Samples	10,324	6,564	5,072^b^
Identified CNVs	14,226^a^	9,186	495,916
Type of CNVs:			
Loss	7,554	5,101	343,489
Gain	6,672	4,085	152,427
Average CNV length (Kb)	3,336	3,014	31
Type of inheritance:			
De novo constitutive	14,501	2,454	
Inherited from normal parent	9,345	1,945	
Inherited from parent with similar phenotype to child	1,345	240	
Unknown	21,946	3,638	

The table shows genotyped patients in DECIPHER database (*All*), the genotyped and phenotyped patients from DECIPHER used in this work (*Cases*) and the healthy individuals from the DGV repository (Control). The first column indicates the distribution of data based on number of individuals, number and type of CNVs and their type of inheritance. ^a^ This is a pre-selection of CNVs from DECIPHER that are potentially pathogenic. ^b^ This number does not correspond to individual samples

### Analysis of phenotypically enriched *loci* (PELs)

We built a patient network, consisting of 6,324 nodes (patients) connected by 89,526 interactions based on the genetic overlapping between patient CNVs, and we calculated some topological parameters (Table 2). The resulting network showed low density, which means that the portion of potential interactions is low compared to the actual interactions in the network, and a high average clustering coefficient, which measures how nodes (patients) tend to cluster together. In addition, we also observed other properties such as a heterogeneous degree distribution, a small average shortest path length, and a high average clustering coefficient of network nodes, available in Additional file 2: Figure S1. These network properties suggest that the patient network appeared to show general features of most large real-world networks in contrast to random networks.

**Table 2:** Topological parameters and properties of patient network

Network parameter	Value
Nodes	6,304
Number of interactions	89,526
Clustering coefficients	0.801
Connected components	5
Network diameter	10
Shortest paths	39,482,458
Average shortest path length	3.706
Average degree	28.403
Network density	0.005

From the patient network, we proceed to study PELs; i.e., significantly enriched genomic *loci* with phenotypes in patient clusters. We designed network-based and enrichment analyses to find genetically and phenotypically related clusters of patients (cliques; see Methods and Fig. 1). In total, 1,042 *locus*-phenotype associations between 487 PELs and 195 enriched phenotypes (HPO terms) were generated. We performed a genome-wide study of CNVs, using as control a dataset of healthy population, to evaluate the significance of genotype-phenotype associations in PELs. A Fisher’s exact test (see Methods) related to previous works was applied [8]. However, our experiment defined genetic associations to exploit patient network relationships, evaluating each *locus* independently instead of using sliding windows as previous works. In addition, redundant and uninformative phenotypes were also removed according to their parent–child relationships (see Methods). Using this systematic approach, we reported 387 specific *locus*-phenotype associations between 336 PELs and 115 different phenotypes (HPO terms; Additional file 3: Table S2). Almost 70 % (336 of 487) PELs were significantly more frequently mutated in patients compared to healthy individuals (*P* < 0.05, Fisher's exact test). We denoted these as pathogenic PELs. Given the nature of collecting pathogenic CNVs in DECIPHER, it is not surprising that we obtained this high percentage (70 %) of potentially pathogenic PELs.

**Fig. 3:**
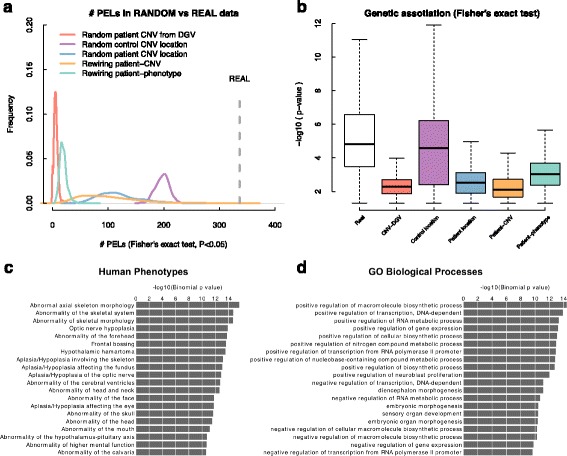
Functional analysis of pathogenic phenotypically enriched *loci*. **a** Distribution of the resulting number of PELs after 1000 randomization experiments, each type of randomization experiment is represented by a colored line tat is described in the legend. The dashed grey line corresponds the number of PELs obtained using the real data. **b** Boxplot of the distribution of *P-values* (*P* < 0.05, Fisher’s exact test) that results from case control analysis. **c** Enrichment of human phenotype in genomic regions related to pathogenic PELS, we used a binomial test from GREAT. **d** Enrichment of biological process in genomic regions related to pathogenic PELS, we used a binomial test from GREAT

To assess whether these *loci* are potentially pathogenic and that our results are not due to chance, we did several randomization analyses with the aim of comparing real and random results. Five different types of randomization analyses were designed using randomized case and control datasets to test if the frequency of detected PELs is lower than real cases (Fig. 3a): (i) we generated random datasets of mutations in patients from random sets of CNVs that were selected from the control dataset (DGV), we used random locations for (ii) patient CNVs and (iii) control CNVs by selecting random genomic regions while keeping CNV length distributions and chromosome frequencies, (iv) the rewiring of the patient-CNV relations, and, finally, (v) the rewiring of phenotype descriptions of patients conserving the phenotype frequencies (see Methods).

We found that the number of PELs identified by using the real data (336) was substantially higher compared to that resulted from the different randomization experiments (Fig. 3a). In addition, the significances (*P-values* < 0.05, Fisher’s exact test) derived from the genetic association study are also higher in real than in randomized datasets (Fig. 3b). The small differences with respect the control dataset with random CNV locations suggest that there is a portion of CNVs in the control population (DGV) that are randomly distributed across the genome, something that might be expected in natural genetics populations (Fig. 3b). Overall these results reveal the existence of a fraction of PELs in DECIPHER that are consistently pathogenic, where both the number of resulting PELs and the median significance of Fisher’s exact test are higher when using real data compared to random datasets (Fig. 3a and b, respectively).

We then studied which annotations from diverse biomedical ontologies are associated with these *loci* using GREAT [29]. It was found that these regions are significantly enriched for human phenotypes (Fig. 3c), reinforcing the probable clinical implication of mutations affecting these genomic regions. In addition, we also found that these PELs are enriched for cis-regulatory domains involved in biosynthetic processes, regulatory elements and embryonic morphogenesis (Fig. 3d). The experimental and functional characterization of these genomic regions might improve our current understanding of the molecular basis of these genomic disorders.

### Pathogenicity of PELs

**Fig. 4:**
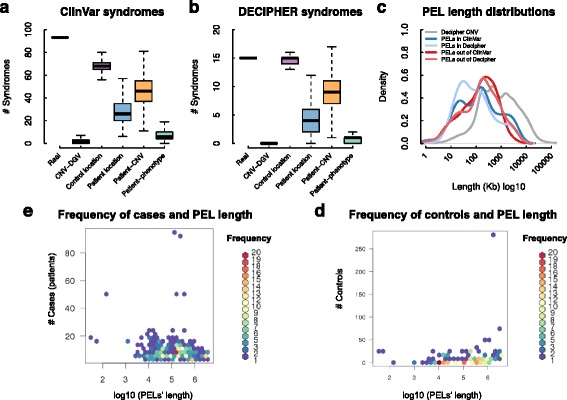
Pathogenicity of phenotypically enriched *loci* (PELs)*.* **a** Boxplot of the distribution of the number of ClinVar syndromes overlapping PELs. Every boxplot represents the resulting number of PELs in real and permuted data; there is only one value for real data. **b** Boxplot of the distribution of the number of DECIPHER syndromes overlapping PELs. **c** Distribution of the PEL lengths overlapping with ClinVar and DECIPHER syndromes, the grey line represent the distribution of the length of the raw patient CNVs from DECIPHER. **d** Relationship between the PEL length and their number of cases (patients), the colors represent the frequency of the observations. **e** Relationship between the PEL length and their number of overlapped controls (DGV), the colors represent the frequency of the observations

With the aim to validate the resulting phenotype-genotype associations, we searched how many pathogenic PELs match with known genomic disorders in ClinVar [30]. For this we selected 2,243 pathogenic or likely pathogenic CNVs associated with any OMIM phenotype and other 75 genomic regions described as DECIPHER syndromes. We then studied if our method retrieves genomic syndromes from ClinVar or DECIPHER. First, we looked for those PELs overlapping known syndrome from both databases (Additional file 4: Table S3 and Additional file 5: Table S4 for ClinVar and DECIPHER respectively) and having the same type of mutation as the described for syndromes (i.e. deletions or duplications). The number of syndromes was determined and real results were compared versus random results (Fig. 4a and b, for ClinVar and DECIPHER respectively). From the real datasets, we counted a total of 93 and 15 syndromes overlapping PELs from ClinVar and DECIPHER respectively. These numbers are higher than the ones obtained from the randomization experiments (Fig. 4a and B), with the exception of those using control CNVs with random locations across the genome. The distributions of the randomizations were similar in ClinVar and DECIPHER but with considerable differences in the number of syndromes (Fig. 4a and b). Although a higher number of known syndromes could be expected, it should be taken into account that DECIPHER includes several cohorts of patients with rare genomic disorders that have not been well characterized. This means that some cohorts of patients that have been already diagnosed for well-characterized syndromes have probably not been sent to the DECIPHER database. To study how the length of PELs could be affecting our approach, we compared their length distributions across the different subset of PELs (Fig. 4c). The average length of PELs overlapping known syndromes is slightly shorter than those classified as potential novel syndromes, and the length of raw CNV from DECIPHER are considerably longer (Fig. 4c). Subsequently, we compared the length of PELs and the number of patient CNVs and control CNVs overlapping these PELs (Fig. 4d and e, for patients and controls, respectively). We observed that the frequency of patients overlapping a PEL is independent to their length (Fig. 4a). This effect could be also explained by the specific cohorts of patient CNVs that are collected in DECIPHER. However, it is observable that the frequency of controls that overlap PELs, despite being very low, increases with PEL length (Fig. 4b). This observation agrees with the random distribution of control CNVs across the genome. Overall, these results evidence that our approach is robust at finding phenotypically enriched *loci* (PELs) from a heterogeneous population of patients of different genomic disorders.

**Fig. 5:**
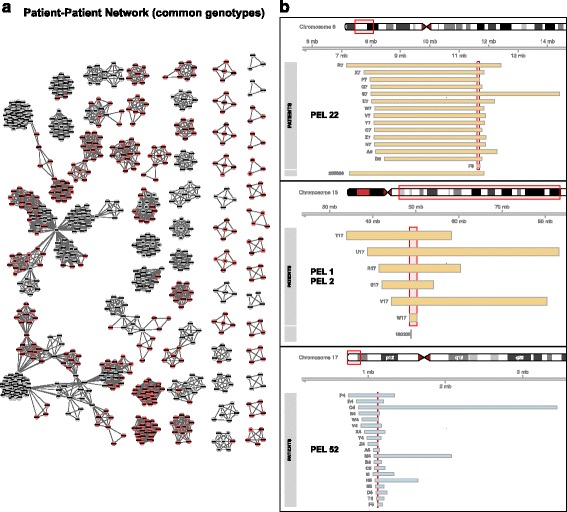
Genetic and phenotypic relationships between patients. **a** Network of the patients associated with the 336 pathogenic PELs. It includes 830 patients (nodes) and 9606 pairwise relationships supported by genotype-phenotype associations (edges). Grey nodes indicate that patient PELs are associated with at least one known syndrome and red nodes indicate that patient PELs do not overlap with any known genomic syndrome. **b** Examples of known and novel PELs. Patients of PEL 52 that coincide with deletions associated with pulmonic stenosis (MIM 265500). PEL 1 and 2 are patients showing coincidences with the 13q14 deletion syndrome in which the most representative clinical feature is retinoblastoma (MIM 180200). PEL 52 is not associated with any known syndrome and it has patients showing split hand (HP:0001171) and duplications in 17p13.3

We also built a patient network from the genotype and phenotype data of individuals related to pathogenic PELs, revealing clusters of patients that correspond to cliques or sets of them. The resulting network represents a map of the most relevant genotype-phenotype associations that we found in the DECIPHER dataset (Fig. 5a). From ClinVar information, we identified patient CNVs with or without an overlap to known genomic disorders (grey and red nodes in Fig. 5a, respectively). A detailed exploration of these clusters of patients revealed that 164 (~50 %) of the pathogenic PELs (see previous section) overlapped pathogenic CNVs in ClinVar, indicating that PELs are potentially related to known genomic disorders (Table 3 and Additional file 5: Table S4). For instance, in Fig. 5b, the PEL associated with the 8p23.1 deletion coincides with the same genomic location as the genetic variants related to pulmonic stenosis (MIM 265500) in ClinVar. In this particular case, 15 out of 21 patients with deletions in this *locus* (Fig. 5b and PEL 22 from Table 3) were annotated with "Malformation of the heart and great vessels" (HP:0002564, *P-value* of the enrichment 8.3E-10), which is the primary cause of pulmonic stenosis. In addition, there was no healthy individual from the control dataset showing a deletion in this *locus*, suggesting a high penetrance of this phenotype associated to this locus (Table 3).

**Table 3:** Phenotypically enriched locus overlapping with phenotypically similar known genomic syndromes

PEL ID	Type*	Chr	Start	Length (Kb)	Phenotype	Cases/Carrier (DGV)	*P* value^a^	P ^b^	MIM^c^
PEL 240	d	1	243981716	12.547	Abnormality of the skull	13/18 (0)	4.50E-08	100	217990
PEL 193	d	1	243786018	126.15	Abnormality of the skull	14/19 (0)	1.30E-08	100	217990
PEL 68	d	1	243981716	12.547	Microcephaly	12/18 (0)	7.40E-11	100	217990
PEL 49	d	1	243786018	126.15	Microcephaly	13/19 (0)	9.20E-12	100	217990
PEL 25	d	1	243981716	12.547	Aplasia/Hypoplasia of the cerebrum	15/18 (0)	1.80E-12	100	217990
PEL 15	d	1	243786018	126.15	Aplasia/Hypoplasia of the cerebrum	16/19 (0)	3.80E-13	100	217990
PEL 70	d	11	31802605	23.093	Aplasia/Hypoplasia affecting the eye	5/8 (0)	1.00E-08	100	106210
PEL 317	d	14	55242483	200.932	Abnormality of the eye	6/6 (0)	1.80E-04	100	248000
PEL 295	d	4	82082415	31.542	Growth abnormality	9/11 (0)	4.20E-06	100	601665
PEL 484	d	6	407031	170.484	Abnormality of the ocular region	10/16 (1)	4.10E-06	30.2	145400
PEL 484	d	6	407031	170.484	Abnormality of the ocular region	10/16 (1)	4.10E-06	30.2	187350
PEL 347	d	6	1612710	15.026	Abnormality of the ocular region	11/17 (0)	1.60E-07	100	145400
PEL 347	d	6	1612710	15.026	Abnormality of the ocular region	11/17 (0)	1.60E-07	100	187350
PEL 156	d	6	407031	170.484	Abnormality of globe location	9/16 (1)	7.90E-08	28	145400
PEL 100	d	6	2371534	63.584	Hypertelorism	8/13 (1)	2.80E-08	25.7	145400
PEL 88	d	6	1612710	357.639	Hypertelorism	9/16 (1)	3.00E-09	28	145400
PEL 58	d	6	1612710	22.698	Hypertelorism	10/17 (0)	3.40E-11	100	145400
PEL 22	d	8	11610366	83.076	Malformation of the heart and great vessels	15/21 (0)	1.00E-13	100	265500
PEL 6	d	8	11610366	83.076	Abnormality of the cardiovascular system	18/21 (0)	8.00E-15	100	265500
PEL 7	d	8	11610366	83.076	Abnormality of cardiac morphology	17/21 (0)	6.40E-15	100	265500
PEL 452	d	X	102585912	9.472	Abnormality of digit	6/8 (0)	3.50E-05	100	108110

^*^ Duplication (D) and deletion (d). ^a^ Adjusted P-values from the Fisher’s Exact test of the case–control analysis. ^b^ P is the penetrance, this table show only those PELs with a penetrance higher than 25 %. The penetrance was calculated as described by Cooper et al. [8, 28]. ^c^ OMIM genomic disorders from ClinVar showing phenotypes that were similar to those found in the respective PEL

Another example is retinoblastoma (HP:0009919, *P-value* of the enrichment 6.7E-16 and 3.7E-15 for PEL 1 and 2 respectively; Additional file 3: Table S2) where 6 out of the 7 cases from the patient dataset belong to the same PEL, consisting on deletions in 13q14.2 (chr13:48,544,437-50,206,474, see Fig. 5b). It has been documented that structural variations in this *locus* are associated with the 13q14 deletion syndrome in which the most representative clinical feature is retinoblastoma (MIM 180200) [31, 32]. However, deletions in this *locus* are frequent in control population (286 samples, Additional file 3: Table S2), suggesting a reduced penetrance for the retinoblastoma phenotype [33] where other factors might be influencing this medical condition. These results indicate that our method is able to identify and prioritize structural variants that are strongly associated with pathological phenotypes.

In addition, several clusters of patients associated with pathogenic PELs that were found not to be apparently associated with known genomic syndromes but significantly enriched for highly specific clinical features such as ectrodactyly, malformations in the heart, defects in atrial septum, and anophthalmia (Table 4). More than 50 % (172 out of 336) of the pathogenic PELs do not overlap with any known genomic disorder in ClinVar so they can be candidates for novel syndromic *loci.* For instance, we detected a cluster of patients showing a severe medical condition that is known as split hand (HP:0001171) with duplications in 17p13.3 (Fig. 5b). The PEL associated with this cluster (PEL 52, *P-value* of 1.1E-13 for Fisher's exact test in Additional file 3: Table S2) shows a very high penetrance for this phenotype, but its patients display a broad spectrum of specific clinical outcomes that are associated with this medical condition. The phenotype "abnormality of the hand" (HP:0001155) was the most enriched HPO term (*P-value* of the enrichment 2.7E-07 for PEL 52 in Additional file 3: Table S2) associated with this PEL (Table 4). *A priori* this cluster of genetically and phenotypically related patients could be considered a novel genomic disorder. Indeed, after reviewing the available clinical literature we found evidence of syndromic presence in micro-duplications spanning this *locus*, related to a previous familiar study with a similar phenotype [34]. We distinguished seven broad domains of phenotypic abnormalities through the examination of the phenotypic relationships between patients from PELs (Additional file 3: Table S2): abnormality of the ocular region, abnormality of the limb bone morphology, abnormality of the skull, abnormality of the face, abnormality of the cerebrum, abnormality of the cardiovascular system and growth delay. Our results show that this approach provides a new tool for the characterization and the study of phenotype-genotype relationships in a systematic genome-wide manner. For instance, it is possible to characterize the pleotropic effects of pathogenic CNVs or to study mutations on different mutated genomic regions that are related to similar phenotypes.

**Table 4:** The novel pathogenic phenotypically enriched locus

PEL ID	Type*	Chr	Start	Length (Kb)	Phenotype	Cases/Carrier (DGV)	*P* value^a^	P ^b^
PEL 3	d	3	181296306	175.931	Anophthalmia	6/9 (0)	1.80E-15	100
PEL 5	d	7	95693340	89.973	Ectrodactyly	7/10 (0)	1.70E-14	100
PEL 4	d	3	181296306	175.931	Abnormality of globe size	8/9 (0)	1.90E-14	100
PEL 4	d	3	181296306	175.931	Aplasia/Hypoplasia affecting the eye	8/9 (0)	1.90E-13	100
PEL 71	d	2	200208169	38.268	Abnormality of the palate	11/19 (0)	2.10E-11	100
PEL 31	d	3	181166306	576	Abnormality of globe size	6/9 (0)	4.10E-11	100
PEL 105	d	2	200208169	38.268	Abnormality of the oral cavity	12/19 (0)	2.50E-10	100
PEL 84	d	15	100019051	189.992	Growth delay	13/18 (0)	2.70E-10	100
PEL 31	d	3	181166306	576	Aplasia/Hypoplasia affecting the eye	6/9 (0)	2.70E-10	100
PEL 128	d	2	200208169	38.268	Abnormality of the mouth	14/19 (0)	1.90E-09	100
PEL 131	d	15	100019051	189.992	Growth abnormality	14/18 (0)	6.40E-09	100
PEL 129	d	15	99057570	65.959	Growth delay	11/16 (0)	8.10E-09	100
PEL 69	d	11	31735689	39.768	Aplasia/Hypoplasia affecting the eye	5/8 (0)	1.00E-08	100
PEL 126	d	2	166091754	49.616	Seizures	10/15 (0)	1.00E-08	100
PEL 175	d	7	112349829	160.71	Delayed speech and language development	12/18 (0)	1.00E-08	100
PEL 78	d	14	29904720	411.94	Aplasia/Hypoplasia of the cerebrum	10/12 (0)	1.50E-08	100
PEL 82	d	14	29904720	411.94	Aplasia/Hypoplasia of the cerebrum	10/12 (0)	1.50E-08	100
PEL 141	d	7	114297499	533.997	Delayed speech and language development	11/15 (0)	4.50E-08	100
PEL 166	d	2	166244769	311.476	Seizures	9/14 (0)	6.00E-08	100
PEL 202	d	15	99057570	65.959	Growth abnormality	12/16 (0)	8.80E-08	100
PEL 152	d	14	29904720	411.94	Microcephaly	8/12 (0)	1.60E-07	100
PEL 159	d	14	29904720	411.94	Microcephaly	8/12 (0)	1.60E-07	100
PEL 216	d	2	200208169	38.268	Abnormality of the palate	7/13 (0)	1.60E-07	100
PEL 385	d	2	200246437	0	Abnormality of the face	16/19 (0)	1.60E-07	100
PEL 137	d	6	76509712	359.49	Joint laxity	5/9 (0)	1.60E-07	100
PEL 390	d	7	112349829	160.71	Neurodevelopmental delay	13/18 (0)	1.60E-07	100
PEL 412	d	13	92065689	29.285	Growth delay	9/17 (0)	2.00E-07	100
PEL 419	d	13	92065689	29.285	Growth delay	9/17 (0)	2.00E-07	100
PEL 436	D	16	3831263	32.469	Abnormality of the face	15/18 (0)	4.20E-07	100
PEL 222	d	2	201936560	57.623	Abnormality of the mouth	10/13 (0)	4.80E-07	100
PEL 222	d	2	200208169	38.268	Abnormality of the mouth	10/13 (0)	4.80E-07	100
PEL 242	d	7	114297499	533.997	Neurodevelopmental delay	12/15 (0)	5.20E-07	100
PEL 114	d	13	48557360	146.432	Abnormality of the globe	8/9 (0)	5.90E-07	100
PEL 250	d	7	119973023	238.728	Delayed speech and language development	9/13 (0)	8.80E-07	100
PEL 123	d	12	66224830	22.517	Short stature	7/8 (0)	1.10E-06	100
PEL 184	d	1	28743173	21.263	Deeply set eye	4/7 (0)	1.90E-06	100
PEL 462	d	1	11270844	47.828	Abnormality of the skull	10/14 (0)	1.90E-06	100
PEL 417	d	2	201936560	57.623	Abnormality of the mouth	9/13 (0)	2.00E-06	100
PEL 330	d	14	29781404	230.359	Seizures	7/12 (0)	2.10E-06	100
PEL 227	d	9	77206264	34.573	Seizures	7/10 (0)	2.10E-06	100
PEL 133	D	2	219965169	9.153	Cutaneous finger syndactyly	3/5 (0)	2.30E-06	100
PEL 116	d	3	181296306	6.681	Abnormality of the ocular region	9/9 (0)	2.50E-06	100
PEL 173	D	2	59105866	181.965	Midface retrusion	3/5 (0)	4.00E-06	100
PEL 120	D	2	59105866	181.965	Strabismus	5/5 (0)	4.40E-06	100
PEL 276	d	7	94174003	41.631	Decreased body weight	4/7 (0)	7.20E-06	100
PEL 329	d	7	95693340	89.973	Abnormality of limb bone morphology	8/10 (0)	1.30E-05	100
PEL 304	d	X	133530468	102.522	Global developmental delay	6/8 (0)	1.50E-05	100
PEL 388	d	10	28842276	86.821	Abnormality of the eyelid	6/8 (0)	1.70E-05	100
PEL 210	D	2	59105866	181.965	Feeding difficulties in infancy	4/5 (0)	1.70E-05	100
PEL 455	d	10	28842276	86.821	Abnormality of the palpebral fissures	5/8 (0)	2.00E-05	100
PEL 251	d	13	41726952	39.763	Abnormal eye morphology	6/7 (0)	2.00E-05	100
PEL 470	d	10	28842276	86.821	Abnormality of the hair	5/8 (0)	2.20E-05	100
PEL 358	d	1	28743173	21.263	Abnormality of globe location	5/7 (0)	2.90E-05	100
PEL 460	d	3	181648378	93.928	Abnormality of the ocular region	7/9 (0)	3.60E-05	100
PEL 338	d	5	170676605	370.857	Abnormality of the cardiac septa	4/6 (0)	3.90E-05	100
PEL 133	D	2	219965169	9.153	Toe syndactyly	3/5 (0)	4.00E-05	100
PEL 454	d	1	177800358	362.172	Short stature	5/7 (0)	4.40E-05	100
PEL 369	d	14	57423809	185.438	Abnormality of the eye	7/8 (0)	4.50E-05	100
PEL 449	d	7	94174003	41.631	Abnormality of the foot	5/7 (0)	4.90E-05	100
PEL 294	d	1	157149743	12.346	Abnormal hair quantity	3/4 (0)	5.70E-05	100
PEL 213	d	1	157149743	12.346	Abnormality of the lip	4/4 (0)	5.70E-05	100
PEL 327	d	19	10640379	140.937	Abnormal genital system morphology	4/5 (0)	7.00E-05	100
PEL 448	d	14	58205713	144.654	Abnormality of the skull	7/8 (0)	8.00E-05	100
PEL 203	d	13	33963658	138.576	Abnormality of the neck	3/3 (0)	8.20E-05	100
PEL 423	d	3	181692255	50.051	Abnormality of the face	9/9 (0)	1.10E-04	100
PEL 324	d	7	94953990	5.573	Growth abnormality	6/6 (0)	2.20E-04	100
PEL 456	D	2	219965169	9.153	Abnormality of the lower limb	4/5 (0)	5.20E-04	100
PEL 361	D	7	106664270	182.398	Strabismus	3/3 (0)	5.40E-04	100
PEL 404	D	7	107527586	136.426	Abnormality of eye movement	3/3 (0)	8.20E-04	100
PEL 407	D	1	113036203	122.933	Abnormality of the palate	3/3 (0)	1.00E-03	100

^*^Duplication (D) and deletion (d). ^a^ Adjusted *P-values* from the Fisher’s Exact test of the case–control analysis. ^b^ This table show only those PELs with a penetrance higher than 100 %. The penetrance was calculated as described by Cooper et al. [8, 28]

### Additive phenotypic effects of pathogenic CNVs

**Fig. 6:**
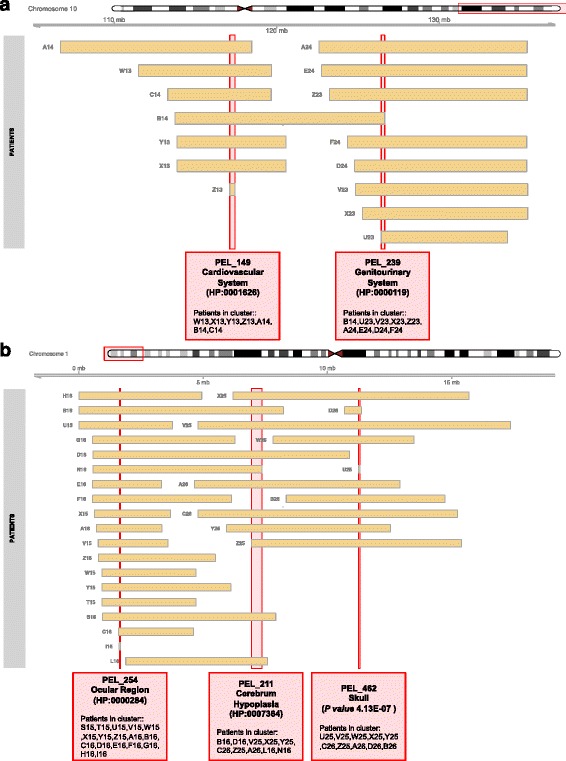
Illustrative examples of additive phenotypic effects of PELs

We observed that the length of CNVs is correlated to complex phenotypic profiles of DECIPHER patients, as shown in Fig. 2a. This complexity is here defined as the number of distinct clinical features that have been observed by a physician in a patient. Thus, it was explored if the length of significant PELs is associated with complex pathogenicity or adds more phenotypes according to the number of different genomic regions that are affected. To illustrate this effect, we analyzed the phenotypic relationships between significant PELs that are in close genomic regions. For instance, deletions in 10q25.13 (PEL 149) and 10q26.13 (PEL 239) are related to different phenotypes such as abnormalities of the cardiovascular system and the genitourinary system respectively (Fig. 6a). Most cases with deletions in 10q25.13 (5 of 7 cases) are associated with malformations of the heart and great vessels, denoting a very specific clinical feature. In addition, cases with deletions in 10q26.13 are related to defects in the genitourinary system (PEL 239 in Fig. 6a). The patient B14 (Fig. 6a) shows both phenotypes and has a deletion that overlaps both genomic *loci* (PEL 149 and PEL 239, Fig. 6a). This example illustrates an additive effect, accumulating specific clinical features according to the extension of structural variants with respect to the genome of reference. This effect is also noticeable for more complex genetic relationships among *loci* of patient CNVs associated with significant PELs as those represented in Fig. 6b. In this case, three different clusters (cliques) of highly interconnected patients were detected, indicating that some individuals are included in more than one cluster or PEL. These different PELs were found to be associated with abnormalities of the ocular region, aplasia/hypoplasia of the cerebrum and abnormalities of the skull (PEL 254, 211 and 462, respectively, Fig. 6b). All patients overlapping these regions from significant PELs show the phenotype if they have the structural variation, except for patient S15 who apparently does not have signs of hypoplasia of the cerebrum. Different PELs associated with the same phenotype (HPO terms) were found located in contiguous or even the same genomic region. In some other cases, distinct PELs were essentially the same clusters of patients except with variations in one or two individuals (they should be considered one PEL). Thus, despite the precise identification of genomic coordinates of individual CNVs being a technological limitation, the wide adoption of next generation sequencing methods by clinical studies may solve the current shortcomings in the array-based CNV data used for this analysis.

## Conclusions

This work presents a combined analysis of network-based approaches, phenotype enrichment and genetic association studies for patient CNVs in the DECIPHER database. A set of methods was developed to identify clusters of patients that are genetically and phenotypically related. The newly developed methods used here have potential usefulness for a wide range of applications, such as prediction of unknown syndromes, characterization of candidate pathogenic structural variants and the identification likely associated phenotypes with a specific *locus*. This procedure could be improved using more specific clinical features of the patients, so physicians should be encouraged to submit detailed phenotype data. This work evidences the need for advancement in consolidated standards and public repositories for genomic and medical records in genomic and personalized medicine.

## Additional files

Additional file 1: Table S1. Collection of DECIPHER patients CNVs, mode of inheritance and phenotypes that have been analyzed from DECIPHER. (XLSX 2440 kb) Additional file 2: Figure S1. Distribution of topological parameters calculated from the patient network based on the overlapping between individual DECIPHER CNVs. (PDF 84 kb) Additional file 3: Table S2. Phenotypically enriched loci (PELs) after the enrichment analysis (hypergeometric test, *P*-values <0.05.) and case–control analysis (Fisher's exact test, *P*-values <0.05). (XLSX 269 kb) Additional file 4: Table S3. Relationship between Phenotypically enriched loci (PELs) and genomic disorders used from ClinVar, OMIM phenotypes caused by likely pathogenic and pathogenic CNVs from ClinVar. (XLSX 64 kb) Additional file 5: Table S4. Relationship between Phenotypically enriched loci (PELs) and genomic disorders used from DECIPHER. (XLSX 59 kb)

## Abbreviations

CNV/s: Copy number variation/s
PEL/s: Phenotypically enriched locus/loci, HPO, human phenotype ontology
DGV: Database of genomic variants
OMIM: Online Mendelian Inheritance in Man
MIM: OMIM identification number
DECIPHER: DatabasE of genomiC varIation and Phenotype in Humans using Ensembl Resources
