# Supplementary material for: Systematic identification of phenotypically enriched loci using a patient network of genomic disorders
Source: BMC Genomics. 2016 Mar 15;17:232. doi: 10.1186/s12864-016-2569-6 (PMC4792099; doi:10.1186/s12864-016-2569-6)

A **Degree distribution**

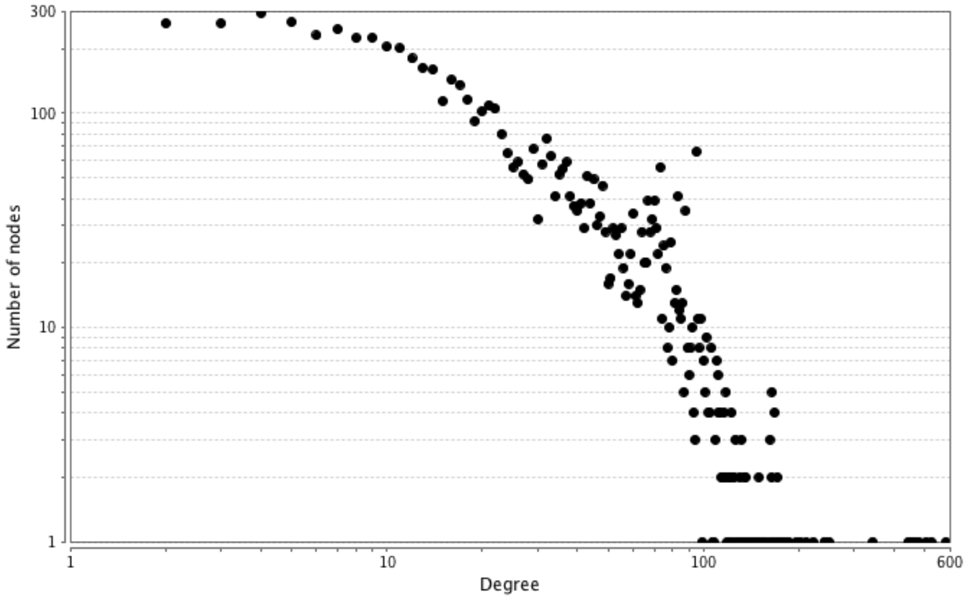

B **Clustering coefficient distribution**

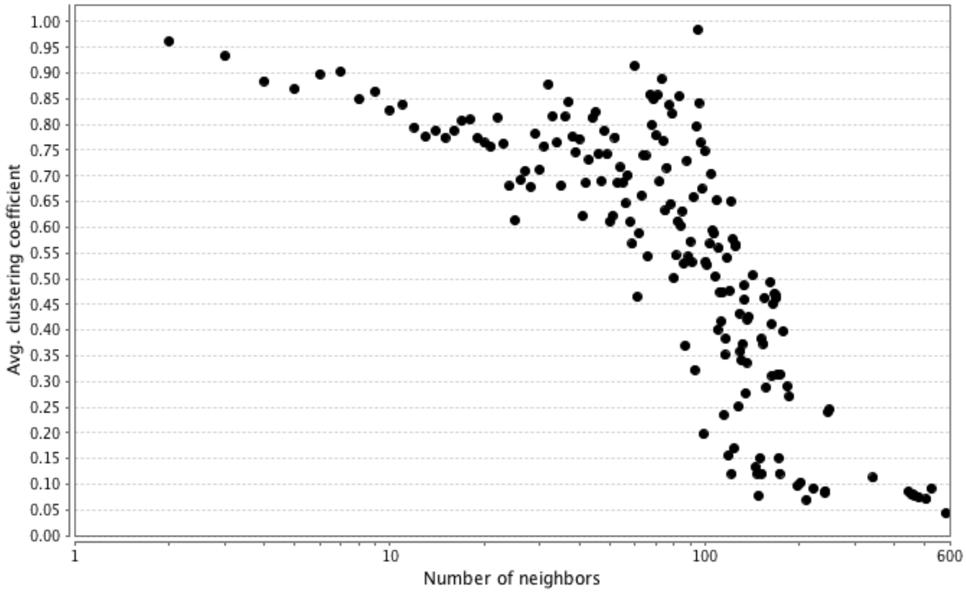

C **Distribution of shortest paths**

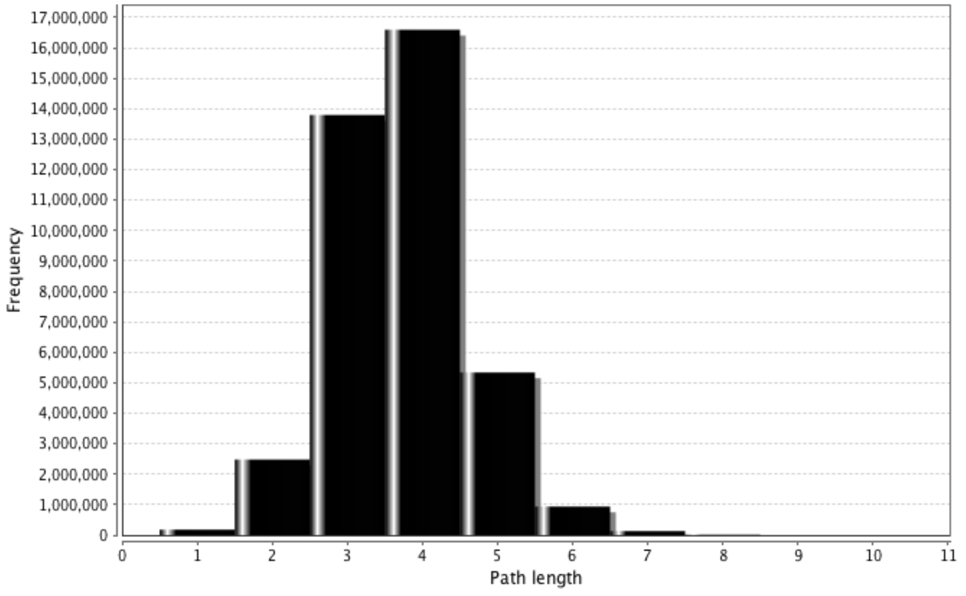

Supplement: Additional file 2: Figure S1. — Distribution of topological parameters calculated from the patient network based on the overlapping between individual DECIPHER CNVs. (PDF 84 kb) [file 12864_2016_2569_MOESM2_ESM.pdf]
